# The Advances in Antipsychotics-Induced Dyskinesia Rodent Models: Benefits of Antioxidant Supplementation

**DOI:** 10.3390/biomedicines13020512

**Published:** 2025-02-18

**Authors:** Uros Velickovic, Dragica Selakovic, Nemanja Jovicic, Marina Mitrovic, Vladimir Janjic, Sara Rosic, Suzana Randjelovic, Dragan Milovanovic, Gvozden Rosic

**Affiliations:** 1 Department of Physiology, Faculty of Medical Sciences, University of Kragujevac, 34000 Kragujevac, Serbia; uvelickovic26@gmail.com (U.V.); rosicsara@gmail.com (S.R.); grosic@fmn.kg.ac.rs (G.R.); 2 Department of Histology and Embryology, Faculty of Medical Sciences, University of Kragujevac, 34000 Kragujevac, Serbia; nemanjajovicic.kg@gmail.com; 3 Department of Medical Biochemistry, Faculty of Medical Sciences, University of Kragujevac, 34000 Kragujevac, Serbia; mitrovicmarina34@gmail.com; 4 Department of Psychiatry, Faculty of Medical Sciences, University of Kragujevac, 34000 Kragujevac, Serbia; vladadok@yahoo.com; 5 Psychiatry Clinic, University Clinical Center Kragujevac, 34000 Kragujevac, Serbia; 6 Department of Emergency Medicine, University Clinical Center Kragujevac, 34000 Kragujevac, Serbia; suzanarandjelovic25@gmail.com; 7 Department of Pharmacology and Toxicology, Faculty of Medical Sciences, University of Kragujevac, 34000 Kragujevac, Serbia; piki@fmn.kg.ac.rs

**Keywords:** antipsychotics, parkinsonism, antioxidants, rodents

## Abstract

After 70 years of clinical practice with antipsychotics in the treatment of some specific serious mental disorders, much information has been accumulated considering their efficiency as a first-line evidence-based schizophrenia therapy, but also on their adverse effects within the range from minor to life-threatening issues. In this paper, we highlight motor impairment as a frequent limiting factor. Despite the diversity of side effects following antipsychotics usage, many of those who suffer share the same pathophysiological background issues, such as oxidative damage, neuroinflammation, apoptosis, and neurodegeneration (observed in the brain regions involved in motor control). The obvious need to solve these limitations is facing restraints in clinical studies due to the ethical issues. Therefore, it seems reasonable to address the importance of preclinical investigations to overcome the adverse effects of antipsychotics. For that purpose, we analyzed the antipsychotics-induced dyskinesia seen in rodent models, with a special focus on attempts to highlight the benefits of antioxidant supplementation. Our analysis has revealed that antioxidant supplementation, with various antioxidant-rich compounds, confirms the clear neuroprotective effects of the therapy of this iatrogenic dyskinesia. Given their accessibility and safety, it seems that the administration of antioxidant-rich compounds in various forms, as an adjuvant therapy, may be beneficial in patients by lowering the risk of secondary Parkinsonism. Also, it seems that the strategy for further investigations in this field of preclinical studies should be standardized and should include more antipsychotics employed in the clinical practice.

## 1. Introduction

After nearly 70 years of the clinical use of antipsychotics in the treatment of various mental disorders, primarily schizophrenia and bipolar disorders, we have seen a range of side effects [1]. Significant adverse reactions associated with antipsychotics, especially at elevated doses, include extrapyramidal motor symptoms (EPSs) such as Parkinsonian symptoms, akathisia, and tardive dyskinesia, which may be regarded as the primary limitation of treatment with these medications [2]. This has encouraged immediate efforts by clinicians and researchers to explore and promote antipsychotics that reduce EPSs. This significant concern has resulted in the classification of antipsychotics based on the risk of EPSs [3]. Numerous investigations have been conducted to simulate the effects of antipsychotic-induced EPSs in clinical trials using animal experimental models, primarily rodents, to address and mitigate the significant limitations of these therapeutics. Due to fact that the complex neurobiology of antipsychotic-induced parkinsonism shares the same underlying mechanisms of neurotoxicity, even with comparable motor impairment features, animal models (including rodent models, as the most frequent [4]) have been considered as reliable preclinical protocols for the evaluation of the pathogenesis, manifestations forms, and possible treatments with conventional anti-Parkinsonism strategies, but also with adjuvant therapeutic approaches, which may reduce nitroso-oxidative stress (as one of the neurotoxic triggers). This study presents recent iatrogenic rodent models for evaluating antipsychotic-induced EPSs, along with their associated pathophysiological mechanisms.

Considering the specific characteristics of each antipsychotic drug, analyzing a common pathophysiological background can be beneficial for improving associated adverse effects, including various forms of dyskinesia. This analysis of extensive literature sources indicates the presence of unified algorithms in the etiopathology of antipsychotic-induced motor dysfunctions [5]. Nitroso oxidative stress in specific brain regions is recognized as a prevalent trigger for antipsychotic-induced motor impairment, alongside causally linked mitochondrial dysfunction [6], neuroinflammation [7], apoptosis [8], and associated neurodegenerative processes [9]. Many researchers identify oxidative stress as a significant dysfunction hallmark with therapeutic potential for treating antipsychotic-induced Parkinsonism-like symptoms. We analyzed the most recent data regarding the potential role of antioxidant supplementation in treating antipsychotic-induced dyskinesia in rodent models. We summarized the current knowledge from the past five years about the benefits of antioxidant use in this field of research.

## 2. Antipsychotics

### 2.1. The Classification of Antipsychotics

Antipsychotic medications, also known as neuroleptics, are used for the treatment and management of symptoms associated with various psychiatric disorders [10]. Neuroleptic medications are classified into two main groups based on distinct criteria, namely, first-generation (typical or conventional) antipsychotics and second-generation (atypical) antipsychotics (Table 1).

While these classifications of antipsychotics lack universal support [11] and the rationale for their application remains unclear [12], they nonetheless provide valuable insights for timely side effect analysis, particularly concerning motor dysfunctions. Additionally, beyond the existing classifications of antipsychotics, there is a clear effort to formally introduce third-generation medications, including aripiprazole, cariprazine, brexpiprazole, and lurasidone [13]. However, this has yet to be adopted by both preclinical and clinical research teams.

**Table 1:** The classification of antipsychotics.

FDA-Approved First-Generation Antipsychotics [14]	FDA-Approved Second-Generation Antipsychotics [15]
chlorpromazine	aripiprazole [13]
droperidol	asenapine
fluphenazine	brexpiprazole [13]
haloperidol	cariprazine [13]
loxapine	clozapine
mesoridazine	iloperidone
molindone	lurasidone [13]
perphenazine	olanzapine
pimozide	paliperidone
thioridazine	quetiapine
thiothixene	risperidone
trifluoperazine	ziprasidone
	lumateperone

### 2.2. The Mechanisms of Antipsychotic Action

The mechanism of action of first-generation antipsychotics involves the postsynaptic blockade of dopamine D2 receptors in striatal and cortical regions, with functional imaging studies indicating that 65% D2 receptor occupancy is necessary for antipsychotic efficacy. However, the nonspecific localization of dopamine binding in regions involved in motor regulation may increase the risk of movement disorders, also known as drug-induced Parkinsonism [16]. The main difference between first-generation and second-generation antipsychotics lies in their mechanisms of action. Second-generation antipsychotics exhibit transient binding to D2 receptors, accompanied by rapid dissociation, as well as antagonistic effects on the 5HT2A receptor and agonistic activity at the 5HT1A receptor. Consequently, second-generation antipsychotics are regarded as having a reduced risk of side effects, including extrapyramidal symptoms [17]. Since the haloperidol-induced dyskinesia is by far the most widely evaluated motor impairment accompanying antipsychotic usage (in the predefined timeframe) in rodent experimental models, it is not surprising that the majority of the confirmed actions were achieved in this model.

To evaluate motor dysfunctions, it is essential to focus on their relationships with the levels of antagonized dopamine D2 receptors in regions critical for normal movement, as these appear to be crucial for extrapyramidal symptoms. Consequently, the risk is greatest when the occupancy of dopamine receptors by antagonists surpasses approximately 85%, an instance typically associated with first-generation antipsychotics [18,19,20], with the most concrete evidence of motor impairment obtained with haloperidol protocols [21]. In contrast, conventional dosages of second-generation antipsychotics, as evidenced in clinical trials, demonstrate D2 receptor occupancy below 85%, which correlates with reduced occurrences of motor dysfunctions [22]. To reduce the stigma associated with the adverse effects of various antipsychotic protocols, particularly dyskinesia, it is prudent to establish an additional guideline based on the motor impairment criteria that predominantly impact patients undergoing treatment with first-generation antipsychotics [23,24], especially after the extended use of these agents [25]. This consideration also extends to individuals receiving second-generation antipsychotics [26], although this is less frequent and again mainly at higher doses [27]. Therefore, it is logical to propose a generalized classification that delineates the risk of motor dysfunctions linked to antipsychotic use, as illustrated in Table 2, based on the summarized findings from clinical trials [28].

**Figure 1:**
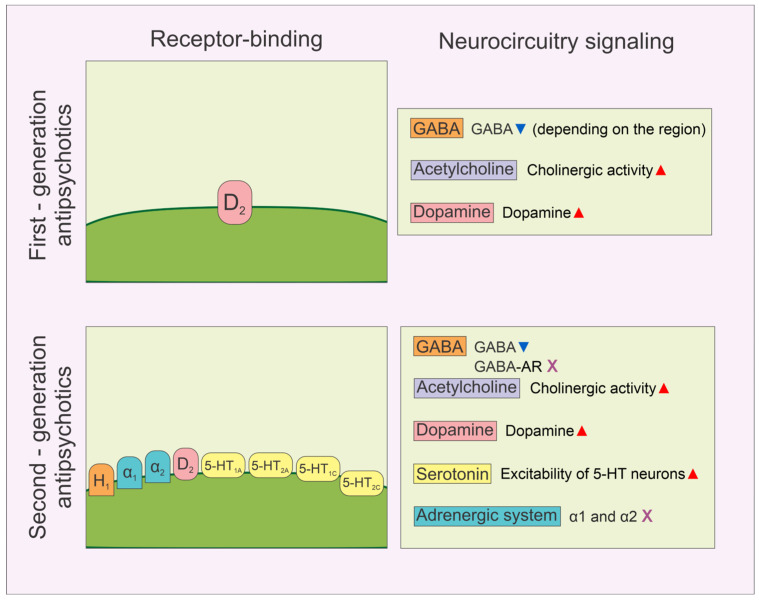
The schematic diagram for overall comparison between the effects of the first- (top panels) and second-generation (bottom panels) antipsychotics on the specific receptors (left panels) and the impacts on neurocircuitry signaling (right panels).

It should be mentioned that both typical and atypical antipsychotics significantly alter neurocircuitry signaling (Figure 1), with specific actions depending on the investigated brain region. Thus, almost all investigated antipsychotics increased cholinergic activity [5] and extracellular dopamine levels [29], while the impacts of the first-generation antipsychotics on the gabaergic system were found to be weaker (decline of GABA levels) when compared to the more pronounced decline in GABA levels accompanied by the GABA-A receptors’ blockade induced by the administration of second-generation antipsychotics [30,31]. In contrast, the effects on serotonin (increased excitability of 5-HT neurons) and the adrenergic system (α1 and α2 receptor blockade) are exclusively seen following the administration of atypical antipsychotics [29].

## 3. The Significance of Preclinical Studies in the Investigation of Antipsychotic-Induced Motor Impairment

In light of rigorous ethical constraints imposed on clinical studies, which notably restrict the potential for investigation focused on preventing and treating motor impairments induced by antipsychotics, animal experimental models emerge as effective and significant research tools. These models exhibit a shared pathophysiological foundation, analogous to clinical trials.

The methodological approach that acknowledges behavioral features specific to rodent models in evaluating antipsychotic-induced dyskinesia was initially proposed by Wadington and colleagues four decades ago. Their study involved the chronic administration of haloperidol over six months, which led to the development of vacuous chewing movement (VCM), serving as evidence of tardive dyskinesia in adult rats [32]. While concentrating on the impairment of orofacial motor functions, certain criteria may be relevant to all manifestations of motor imbalance. For instance, there are prolonged effects after the discontinuation of the drug, whereby dyskinesia emerges spontaneously and without purpose following chronic administration of the drug, among other considerations. In summary, this particular spectrum of motor impairment has been characterized as a tardive syndrome, an iatrogenic movement disorder resulting from the administration of drugs that inhibit dopamine receptors [33].

In compliance with the established common etiopathological background that involves various mechanisms (oxidative damage, neuroinflammation, apoptosis, neurodegeneration, etc.), alongside shared motor algorithms in response to antipsychotics, there has been a growing trend of utilization of animal experimental models aimed at enhancing understanding and exploring novel therapeutic strategies. Given their accessibility and affordability, rodent models are the predominant choice for exploring potential causal and adjuvant therapeutic strategies related to antipsychotic-induced dyskinesia, including antioxidant supplementation.

## 4. Antioxidant-Based Strategies Targeting Antipsychotic-Induced Dyskinesia in the Rodent Models

Faced with the disruption of oxidative equilibrium and the subsequent impact on other intracellular effective mechanisms, numerous efforts have been made to evaluate the potential use of antioxidant supplements in non-specific responses to antipsychotic actions via various motor dysfunctions. The results of numerous clinical trials highlight the causal connection between the antipsychotic drugs and oxidative stress. Interestingly, there is no uniform explanation for this relationship, since the first-generation antipsychotics showed pro-oxidant effects [34], while the usage of second-generation antipsychotics resulted in the opposite effects, suggesting that at least part of their neuroprotective effect may be due to antioxidant action [35]. In order to allow for a comparison with data obtained in clinical trials, here we provide an overview of the findings obtained on antipsychotic-induced dyskinesia in rodent models with antioxidant-based strategies over the last five years. A careful review of the existing literature regarding antioxidant supplementation in antipsychotic-induced parkinsonism within rodent models was undertaken by consulting articles from three distinct databases: Web of Science, PubMed, and SCOPUS. The following keywords were employed for the literature research: antipsychotics (each particular drug), Parkinsonism (dyskinesia, motor impairment, catalepsy), and rodents (rats, mice). The criteria for inclusion and exclusion were the presence of antioxidants and the absence of antioxidant protocols. The inquiry was conducted independently from November 2024 to January 2025 by three reviewers (UV/DS/GR) and was restricted to studies published up to and including the year 2020. We restricted our research to articles published in academic journals; thus, unpublished dissertations, conference abstracts, and other data were eliminated. The principal limitation of this study is the lack of data considering the animal models of Parkinsonism following second-generation antipsychotics administration, especially considering the coinciding oxidative damage and/or antioxidant supplementation as a therapeutic strategy. Indeed, second-generation antipsychotics are considered less harmful by means of motor impairment [36], but still, this adverse effect is present, and therefore there is an obvious necessity for further investigations on this issue, including preclinical studies. Interestingly, the new experimental protocols that should be performed may also be focused on the findings that second-generation antipsychotics (risoeridone, clozapine) showed antioxidant potentials and neuroprotective properties via a mechanism that involves microglial response modulation [37].

Mezzomo and colleagues conducted an investigation into the effects of haloperidol (1 mg/kg/day i.m., for 28 days) on male Wistar rats, aiming to induce dyskinesia [38]. This was verified through behavioral evaluations that quantified vacuous chewing movements (VCM) and assessed locomotor activity, demonstrating a positive response through both parameters. The administration of haloperidol produced a significant pro-inflammatory response in the striatum, characterized by increased levels of pro-inflammatory cytokines IL-1β, TNF-α, and IL-6 compared to the control group. Interestingly, the simultaneous oral administration of isoflavones (80 mg/kg) was sufficient to alleviate both motor dysfunctions and the neuroinflammatory response to haloperidol (except IL-6) (Table 3). Wang and colleagues’ subsequent trial employing antioxidant supplementation [39] verified the beneficial effects of this therapeutic approach in the treatment of haloperidol-induced dyskinesia. Once more, the parenteral administration of haloperidol (1 mg/kg/i.p. over a 21-day period) led to the emergence of tardive dyskinesia orofacial manifestations, characterized by heightened VCM and tongue protrusion. These symptoms manifested alongside a pro-inflammatory response, evidenced by elevated levels of TNF-α, IL-1β, and IL-6, as well as a pro-apoptotic effect indicated by an increase in caspase-3 within the striatum of the rats. Furthermore, the administration of haloperidol led to pro-oxidative effects, evidenced by an increase in lipid peroxidation as indicated by the index of lipid peroxidation expressed as thiobarbituric acid reactive substances (TBARS) and nitrite levels, alongside a reduction in antioxidant capacity marked by diminished levels of gluthatione (GSH), superoxide dismutase (SOD), and catalase (CAT) activity. Additionally, there was a notable deprivation of neurotransmitters, characterized by decreased concentrations of dopamine, norepinephrine, serotonin, and 5-hydroxyindoleacetic acid, while levels of 3,4-dihydroxyphenylacetic acid and homovanillic acid were found to be elevated in the rat striatum (Table 3). The presented neuroprotective isoflavones action is in line with the results obtained in an in vitro study on the prevention of oxidative damage and its influence on monoamine oxidase activity [40], as well as the relation to other neuroprotection mechanisms [41].

Higher doses of naringin (300 mg/kg per os for 21 days), a bioflavonoid with antioxidant and anti-inflammatory properties [61], when administered simultaneously with haloperidol, successfully leveled control values for altered motoric and almost all biochemical parameters induced by haloperidol administration. The other group of authors confirmed the benefits of antioxidant supplementation in the treatment of haloperidol-induced orofacial dyskinesia in rats, in the study where Chen and colleagues [42] investigated the therapeutic potentials of vitexin, a C-glycosylated flavone present in various medicinal herbs, which has been previously proven to possess antioxidant, anti-inflammatory, and neuroprotective properties [62] within this iatrogenic model of orofacial dyskinesia (Table 3). The administration of haloperidol (1 mg/kg i.p., 21 days) to Wistar rats resulted in motor dysfunction, as evidenced by an increase in the frequency of vacuous chewing movements and tongue protrusion. The implemented protocol induced numerous alterations in the equilibrium of brain tissue, characterized by increased nitrosative stress (increased nitrite concentrations) and oxidative stress (elevated lipid peroxidation), a reduction in antioxidant capacity (decreased levels of GSH, SOD, and CAT), clear mitochondrial dysfunction (reduced activity of succinate dehydrogenase, total ATPase, NADH–cytochrome c reductase (complexes I–III), and succinate–cytochrome c reductase), neuroinflammation (elevated levels of pro-inflammatory markers—TNF-α, IL-1β, and IL-6), and apoptosis (increased caspase-3 activity) within the rat striatum. Nonetheless, the simultaneous administration of vitexin at a higher applied dose (30 mg/kg i.p.) proved sufficient for preventing both the behavioral and biochemical indicators of haloperidol-induced orofacial dyskinesia. It is worth noting that, while a higher dose of vitexin resulted in a complete recovery of all parameters, this protocol can be regarded as safe due to a documented favorable safety profile in both animal and human studies [63]. A more comprehensive analysis of this investigation may reveal that the neuroprotective effect of vitexin was mediated through the nuclear factor erythroid-2-related factor 2 pathway. This was achieved by utilizing trigonelline, an inhibitor of the nuclear factor erythroid-2-related factor two-mediated pathway.

Given that quercetin has been documented to exhibit neuroprotective properties, particularly in alleviating damage to striatal neurons and consequently improving motor functions [64], Rafic and colleagues conducted a study to evaluate its potential impact on Parkinsonism in rats treated with haloperidol [43]. Following a four-week haloperidol (5 mg/kg, i.p.) treatment, the animals subjected to the extended protocol exhibited numerous behavioral adverse effects (decreased locomotor capability as measured in the home cage, open field, inverted screen, and beam-walking tests), including a significant decrease in water and food intake as well as body weight. Analyses of brain tissue samples have confirmed the prooxidative effects of haloperidol, evidenced by an increase in oxidative damage (MDA) alongside a reduction in antioxidant capacities (SOD, CAT, GPx, GSH). Indeed, chronic quercetin supplementation (100 mg/kg, orally for 21 days) was sufficient to reverse all of haloperidol’s behavioral and biochemical adverse effects (Table 3).

Sirajo and colleagues applied the initial knowledge that the interaction between vitamins D3 and A is the result of an allosteric heterodimer complex formed between the vitamin D receptor and the retinoid X receptor [65], and that motor deficits in PD models can be ameliorated by vitamin D3 receptor stimulation with a vitamin D3 supplement [66] to evaluate Parkinsonism in a haloperidol mice model [44], which shows significant similarity with the rat models in terms of doses (1–10 mg/kg), route (mainly intraperitoneal), and duration (3–4 weeks). Mice given haloperidol (10 mg/kg, i.p.) for three weeks experienced significant motor impairment, including locomotor asymmetry (cylinder test) and muscular strength (paw grip endurance test). This was accompanied by increased oxidative stress (increased MDA, decreased SOD) and cellular toxicity (increased LDH) in brain tissue samples. However, both the simultaneous oral supplementation of vitamin D3 (800 IU) and vitamin A (1000 IU) and their combination (800 IU and 1000 IU, respectively) for 21 days were sufficient to reduce the analyzed indications of haloperidol toxicity. The applied methods had similar effects on neurodegeneration in the motor complex samples that were investigated. It is noteworthy that bromocriptine, a standard agent used in the management of PD at a dosage of 5 mg/kg, produced effects nearly equivalent to those observed with vitamin supplementation (Table 3).

As nanoparticles showed target delivery to the brain, enhanced bioavailability, and ensured the protection of the drug from degradation with a reduction in side effects and dose reduction [67], while cerium oxide has shown the ability to bind oxygen irreversibly (allowing effective ROS scavenging [68] and to display catalase and superoxide dismutase mimicking capabilities [69]), Khan and coworkers [45] used both named advantages to apply cerium oxide nanoparticles intranasally in rats with haloperidol-induced Parkinsonism (Table 3). Thus, the author applied a haloperidol (0.23 mg/kg, i.p., for 14 days) treatment to male Wistar rats, with or without supplementary protocols of oral levodopa solution (4.97 mg/kg), alone and simultaneously with cerium oxide nanoparticles (intranasally, 6 mg/kg). The haloperidol protocol impaired motor and exploratory behavior in open field and pole tests. It also increased oxidative damage (increased TBARS, decreased CAT, SOD, and GSH), inflammation (increased IL-6 and TNF-α), and dopamine levels in brain tissue samples. A histopathological investigation of a specific brain region essential to behavior regulation (the CA1 region of the hippocampus) revealed neuronal damage indications after haloperidol administration. The levodopa treatment was successful in reducing parameters that show haloperidol-induced neurotoxicity, but these positive benefits were enhanced by the addition of cerium oxide nanoparticles, suggesting their potential neuroprotective role.

Another attempt to treat haloperidol-induced PD with antioxidant-rich compounds was conducted by Barroso-Hernández and colleagues [46]. Based on observations indicating ω-3 PUFA levels, particularly eicosapentaenoic acid and docosahexaenoic acid, corresponding with multiple mental processes in humans [70] and rodents [71], the effects of ω-3 PUFA-rich algal oil were examined on the basis of this iatrogenic model of Parkinsonism. Male Wistar rats were given haloperidol (1.5 mg/kg, i.p., for 14 days), which reduced locomotion (the open field test), decreased D2 dopamine receptor expression in medial brain areas, and altered the fatty acid profile of brain tissue in frontal and occipital cortex samples. The prolonged supplementation of algal oil rich in ω-3 polyunsaturated fatty acids (4 g/kg/day of the algae oil supplement, equivalent to 300 mg of ω-3 PUFA/kg/day), performed 6 weeks prior and two weeks concurrently with haloperidol, reversed all haloperidol-induced neurotoxic manifestations in this study (Table 3).

Other antioxidants derived from plants, such as Rhinacanthin-C (RC, a 1,4-naphthoquinone ester), an active compound extracted from R. nasutus leaves, were employed here, as well as in antipsychotics-induced dyskinesia [47]. This compound has been studied for a variety of therapeutic benefits, including antioxidant, anti-inflammatory, and neurodegenerative disorders such as Alzheimer’s disease and Parkinson’s [72]. Albino mice of both sexes were treated with haloperidol (1 mg/kg for 25 days), which was followed by behavioral testing that revealed the increased haloperidol-induced reduction in the mice’s ability to correct an unusual posture (the block test), their sensorimotor coordination (the rotarod test), and their motor coordination and balance (the beam-walking test), as well as a decline in total locomotion (the locomotor activity test using an actophotometer) and exploratory activity (the hole–board test). Haloperidol’s behavioral features were accompanied by increased oxidative and nitrosative stress (increased MDA and decreased SOD, CAT, and GSH; increased nitrite levels, respectively), neurotransmitter imbalance (decreased dopamine, 5-HT, and NE levels), and neurodegeneration (disorganization of neuronal cells) in brain tissue samples. The simultaneous treatment of RC (5, 10, and 20 mg/kg, dissolved in vegetable oil, for 25 days) was sufficient to prevent all estimated indications of haloperidol-induced dyskinesia, with no evident dose dependence (Table 3).

Due to the numerous adverse events associated with the use of standard agents that effectively reverse the symptoms of PD by activating central dopaminergic receptors, such as levodopa, carbidopa, selegiline, amantadine, pergola, and orphenadrine [73], an effective and safe complementary herbal curative agent remains of clinical interest. With the ability to cross the blood–brain barrier [74] and a wide range of optimal pharmacological properties (anti-inflammatory, antioxidant, and anti-aging) [75], icariin, a flavonoid derived from Herba Epimedii, was evaluated to prevent the haloperidol model of Parkinsonism by Sabry’s research team [48]. Male albino Wistar rats were administered haloperidol (1 mg/kg i.p., for two weeks) alone or in combination with L-DOPA (30 mg/kg orally) or icariin (100 mg/kg orally). Behavioral testing was conducted to measure locomotion activity (the open field test) and motor coordination (the rotarod test), and it was demonstrated that motor deprivation caused by haloperidol was reduced by L-DOPA, but even more so after the icariin treatment. A similar approach was observed for oxidative stress markers, where both preventative treatments (L-DOPA and icariin) diminished haloperidol-induced oxidative damage and restored neurotransmitter levels that had previously been reduced by haloperidol action (the effects were greater with icariin). Finally, a histopathological analysis of midbrain region samples revealed severe degenerative changes caused by haloperidol treatment (shrunken neurons, bleeding, vacuolations, and Lewy bodies), which were reversed by both L-DOPA and icariin administration (Table 3). Altogether, according to the results of this study, it seems that the employment of adjuvant antioxidant supplementation with icariin may be beneficial, along with the primary therapeutic approach with L-DOPA, in mitigating the patophysiological background of haloperidol-induced Parkinsonism.

Cost–benefit analysis is vital, involving the important clinical need to develop alternative pharmacotherapies derived from natural sources to prevent and treat the detrimental consequences of PD at a low cost. In this regard, Saleem and coworkers [49] made a significant contribution by estimating curcuminoid compositions against haloperidol-induced PD in a rat model. A curcuminoids-rich extract (binary and ternary inclusion complex formulations, 15–30 mg/kg/day, orally), L-dopa, and carbidopa (100 and 25 mg/kg/day, orally, respectively) were given concomitantly with haloperidol (1 mg/kg/day, i.p.) for three weeks (Table 3). Overall, the curcuminoids formulation protocol was effective in reducing haloperidol-induced neurotoxic effects (behavioral disorders, antioxidant protective mechanisms, and neurotransmitter imbalance), as well as histopathological patterns (neuronal loss, pigmentation, and the formation of Lewy bodies).

Lawal’s research team was also motivated to evaluate the therapeutic potential of antioxidant-rich medicinal plants used to treat typical antipsychotic-induced dyskinesia [50]. They investigated the potential influence of *Datura metel* extract, which contains active phytoconstituents with confirmed neuropharmacological potential, based on its anticholinergic, anti-inflammatory, and antioxidant properties [76]. Interestingly, the potential of this natural antioxidant compound has already been confirmed in the treatment of PD, yet from a different source (organophosphate poisoning [77]). This investigation was conducted on Wistar albino mice (both sexes) using a haloperidol protocol (5 mg/kg/day, infusion) with or without the concurrent administration of neuroprotective agents, as follows: *Datura metel* extract in three doses (50, 100, 200 mg/kg/day, per os), and levodopa (30 mg/kg/day, i.p.). The effects were assessed in two cross-sections—acute (on the first day of administration) and subchronic (on the seventh day of administration) to compare early and delayed responses to the applied protocols. The rotarod test and the pole test demonstrated a significant decrease in motor characteristics in haloperidol-treated mice, which occurred immediately but persisted for one week. The influence of neuroprotective protocols on haloperidol-induced dyskinesia was successfully averted by the simultaneous administration of both *Datura metel* extract and levodopa, with the highest dose of plant extract having the most noticeable effect. Further investigation (molecular docking) allowed the authors to hypothesize that the synergistic inhibition of alpha-synuclein and dopa decarboxylase by *Datura metel* phytoconstituents (particularly atropine and scopolamine) could be causally related to this neuroprotective effect (Table 3).

Cucurbita pepo, a plant found throughout Pakistan, is thought to be a rich source of compounds (terpenoids, cucurbitacin glycosides, flavonoids, and cardiac glycosides) with previously reported anti-inflammatory, antidiabetic, antibacterial, anti-ulcer, antioxidant, antitumor, and antihyperlipidemic properties [78]. Cucurbita pepo seeds also contain omega 6 and 3 fatty acids, L-tryptophan, and γ-tocopherol, which are relevant to this topic because they have been shown to reduce Parkinsonian symptoms [79]. Salem and colleagues conducted a study on albino rats of both sexes [51], in which haloperidol (1 mg/kg/day, i.p. for 21 days) was given alone, as well as in combination with Cucurbita pepo extract at various doses (200, 400, and 600 mg/kg/day, per os for 21 days) and levodopa and carbidopa (100 and 25 mg/kg/day, orally, respectively). A significant variety of behavioral tests were used to assess the primary clinical manifestations of the antipsychotics’ harmful effects (Table 3). The behavioral manifestations of rigidity quantified in the catalepsy test were consistent with the results obtained in the open field test (decline in exploratory activity, grooming, and locomotion), restricted muscle strength performance (hang test, ladder climbing, and swimming test), and extent (foot printing test), confirming the variety of forms of haloperidol-induced dyskinesia. All neuroprotective methods used showed considerable improvements in motor function. However, it should be noted that the most positive effect was shown with the largest dose of plant extract, which nearly equaled the effect of the conventional protocol (levodopa and carbidopa). The haloperidol procedure also caused brain nitrosative (elevated nitrite levels) and oxidative stress (increased lipid peroxidation), as well as a decrease in antioxidant capacity (lower GSH, SOD, and CAT), which were restored by neuroprotective protocols in the same way as behavioral abnormalities were. Estimated monoamine levels in brain tissue samples demonstrated the dose-dependent potential of Cucurbita pepo extract, with nearly the same intensity as the standard therapy protocol (levodopa and carbidopa) and following the same algorithm as is used in restoring brain tissue architecture affected by the haloperidol protocol, as observed via histopathological analyses of brain tissue samples. Given the numerous side effects associated with typical medication, it appears probable that safe antioxidant treatments could be used as a clinical strategy in the treatment of PD’s side effects.

Brassica juncea extract was also used to test the efficacy of herbal components in treating PD symptoms [52]. Previous research has shown that Brassica juncea extract has antioxidant and free radical scavenging activity, which is most likely related to its high concentration of phenolic and flavonoid components [80]. This study used rats of both sexes treated with the most common haloperidol treatment (1 mg/kg/day, i.p. for 21 days) to produce motor impairment. The expected neuroprotective impact was calculated using a conventional protocol (L-Dopa 100 mg/kg/day and carbidopa 25 mg/kg/day, orally) and, as a prospective treatment strategy, Brassica juncea extract (200, 400, and 600 mg/kg dosages orally for 21 days). Symptoms of haloperidol-induced neurotoxicity were confirmed in the battery of behavioral tests (the catalepsy test—increased muscle rigidity; horizontal bar—decreased motor coordination; hang test—decreased muscle strength), as well as via the increased oxidative stress markers (increased lipid peroxidation index, accompanied with a decline in antioxidant capacity—lowered GSH, SOD, and CAT), neurotransmitter disbalance (dopamine levels decrease with an augmentation of monoamine oxidase B activity), and the histological confirmation of the depletion and vacuolization of dopaminergic neurons in the brain tissue samples (Table 3). The described adverse effects of the haloperidol protocol were significantly reduced by both B. juncea extract (the strongest effect was observed with the highest dose) and the standard protocol, implying that plant-based antioxidant compounds can be used as an equally potent (when compared to standard protocols for treating PD symptoms), safer, and less expensive treatment.

Ashhar and associates made a substantial contribution to the evaluation of prospective PD symptom treatment, based on pathophysiological grounds involving severe oxidative damage, by combining two drugs with demonstrated antioxidant characteristics [53]. The authors combined glutathione, a thiol-reducing agent in the brain that may interfere with GSH depletion in the substantia nigra (whose intensity correlates with the severity of PD [81]), and bromocriptine mesylate (BRM), a dopamine receptor agonist, with clinical use in treating motor impairment associated with levodopa treatment for long-term treatment in PD. Furthermore, BRM has antioxidant and antiapoptotic properties that may be associated with the etiology of PD [82]. Adult Wistar rats of either sex were treated with haloperidol (0.23 mg/kg/day, i.p., for 14 days) alone and in a BRM plus GSH combination (0.21 and 61.67 mg/kg/day intranasally, for 14 days, respectively), delivered as a solution or nanoemulsion. Exclusive criteria for the neuroprotective effects of GSH and BRM were developed using the values acquired from a battery of behavioral tests. Thus, the findings of the forced swimming test indicate decreased swimming time and increased immobility time after conducting the haloperidol protocol, whereas the locomotor activity test revealed a substantial drop in total locomotor activity. The rotarod and akinesia tests showed decreased muscular coordination and slower movement initiation after haloperidol treatment (Table 3). These results are consistent with the haloperidol-induced increase in latency time in the catalepsy test. Both versions of the BRM + GSH combination effectively reduced motor impairment symptoms associated with haloperidol treatment. However, it is worth noting that the nanoemulsion form had a significantly stronger effect than the solution.

Lauric acid (LA) is a medium-chain fatty acid found in coconut oil with a high antioxidant potential [83]. This was most likely the motivation of Zaidi and colleagues [54] investigating the potential role of this compound in haloperidol-induced PD, which may be caused by oxidative damage [84]. The experimental protocol included giving Wistar rats haloperidol (1 mg/kg/day i.p., for 14 days) and, after PD induction, standard anti-PD treatment with levodopa (30 mg/kg/day, orally, for 35 days) and lauric acid in two different doses (Table 3). Various rodent behavior patterns were examined to determine the initial effects of haloperidol. The findings reveal that haloperidol-induced weight loss was associated with lower food intake and motor ability (rotarod and beam-walking tests). Haloperidol treatment led to motor impairment, as well as elevated pro-oxidative (increased MDA, NO levels, and decreased SOD levels) and pro-inflammatory responses in the striatum, including increased TNF-α, NF-κB and IL-8, and decreased IL-4. All neuroprotective protocols were beneficial in terms of reducing haloperidol’s adverse effects. However, it should be noted that the highest response was obtained with a larger LA dose. Previous research has indicated the safety of LA at doses of up to 2000 mg/kg body weight in female rats [85], which should be considered when comparing various neuroprotective drugs.

Betaine (BT) is a non-toxic, safe dietary nutritional supplement that is commonly utilized by both people and animals [86]. BT is widely known for its antioxidant, anti-inflammatory, and anti-apoptotic properties, as well as its ability to inhibit cancer growth [86,87]. These properties appeared promising to Tseng and collaborators, who investigated the potential use of betaine in the field of haloperidol-induced motor dysfunctions [55]. The investigation was conducted on male Wistar rats treated with haloperidol (1 mg/kg/day i.p., for 21 days), while the neuroprotective impact of betaine was assessed in two doses (30 and 100 mg/kg/day i.p., for 21 days). Haloperidol’s effect on motor performance was confirmed by orofacial dyskinesia, which occurred concurrently with increased indicators of nitrosative and oxidative stress (increased nitrite level and TBARS, decline in GSH, SOD, and CAT), mitochondrial dysfunction (decline in SDH, total ATPase, NADH-cytochrome C reductase (complex I-III), and succinate-cytochrome C reductase (complex II-III)), neuroinflammation (TNF-α, IL-1β, and IL-6), and an apoptotic marker (caspase-3) in the striatum. Betaine coadministration significantly reduced every parameter of haloperidol-induced neurotoxicity, with a greater effect indicated at higher doses (Table 3).

Plant-based foods are widely known for containing high levels of phenolic compounds such as p-coumaric acid. This hydroxycinnamic acid-family phenolic acid, which can be found (in various forms) in plants and mushrooms [88], has been shown to have numerous beneficial effects, including antioxidant, anti-inflammatory, antimutagenic, antiulcer, antiplatelet, and anticancer properties, as well as the ability to reduce neuronal injury, anxiety, gout, and diabetes [89,90]. Pathan and colleagues used male Swiss albino mice subjected to three different doses (50, 75, and 100 mg/kg/day orally, for 21 days) in conjunction with haloperidol (1 mg/kg/day i.p.) to evaluate the possible new indication for p-coumaric acid use [56]. Haloperidol-induced catalepsy was confirmed in this experimental design by increased indicators of catalepsy and locomotor activity, as well as decreased motor coordination, due to either haloperidol auto-oxidation or degradation by an oxidase [91], or the chronic blockage of dopamine D2 receptors in nigrostriatal neurons [92]. All indications of haloperidol-induced motor impairment were successfully mitigated by p-coumaric acid coadministration; however, there was no obvious dose dependence. The motor dysfunctions caused by the haloperidol regimen were followed by oxidative imbalance in brain samples, which manifested as increased lipid peroxidation (MDA) and decreased antioxidant capacity (Table 3). Again, antioxidant supplementation was sufficient to restore oxidative equilibrium and dopamine levels in a dose-dependent manner, indicating that p-coumaric acid may play a potential role in the treatment of antipsychotic-induced motor impairment.

Selegiline, a monoamine oxidase inhibitor approved by the US FDA [93] for the treatment of PD symptoms at a dose of 10 mg/day, was used in the study to evaluate the possible augmentation of its efficiency in the animal experimental model of iatrogenic Parkinsonism [57]. The primary intervention was the addition of quercetin, another confirmed neuroprotective agent for the treatment of PD symptoms [57], followed by a lipid nanocarrier, a lipid-based nanoformulation, that provides a large surface area to drug particles for better absorption, promoting drug distribution in the brain via the oral route [94]. Albino Wistar rats of either sex were given haloperidol (0.225 mg/kg/day, i.p., for 15 days), whereas the other groups were given selegiline (0.16 mg/kg/day) alone or in combination with quercetin, and the same combination was supplied as a lipid-based nanoformulation (Table 3). Following the completion of the haloperidol protocol, behavioral testing revealed decreased muscle coordination (the rotarod test), increased movement latency on the horizontal bar, slower movement initiation (the akinesia test), and decreased total motor performance in the forced swimming test. Selegiline reduced haloperidol-induced motor impairment. This impact was amplified by the addition of quercetin, but the nanoformulation provided only minor benefits based on predicted behavioral parameters. The same result was observed when DPPH’s decreasing property was evaluated. Indeed, further efforts should be made to strengthen the utility of numerous medications in anti-PD therapy.

Although several major clinical trials failed to establish the effects of coenzyme Q10 (CQ10) [95,96], other clinical [97] and preclinical [98] investigations found that CQ10 treatment reduced the progression of PD. Starting with the fact that the reduced form of CQ10 has been shown to have numerous protective properties, such as antioxidant activity (beneficial in the management of neurodegenerative diseases), Onaolapo and colleagues conducted an investigation to investigate the protective effects of CQ10 supplementation, alone or in combination with levodopa-carbidopa treatment, in chlorpromazine-induced Parkinsonism-like dyskinesia in male mice [58]. The animals were treated with chlorpromazine (5 mg/kg/day i.p., for 21 days), whereas neuroprotective procedures were examined concurrently using a CQ10-supplemented diet in two doses (60 and 120 mg/kg of feed) and levodopa-carbidopa (10 mg/kg/day, orally). The results of the behavioral investigation revealed decreased locomotion, exploratory activity and grooming (open field test), decreased spatial working memory parameters (radial arm and Y maze), and increased immobility time (catalepsy test) in chlorpromazine-treated animals, confirming the motor disbalance caused by this antipsychotic medication (Table 3). However, the results from the raised plus maze test do not correspond to the results for other motor aspects of chlorpromazine effects. The motor imbalance caused by chlorpromazine was associated with the significant confirmation of oxidative damage (increased MDA, decreased SOD), increased NO, and dopamine depletion in the brain homogenate. Overall, the conditions of treated animals were considerably influenced by a decrease in food intake and body weight, which resulted in higher mortality. All neuroprotective procedures used were effective in reducing chlorpromazine’s neurotoxic effects. However, it should be noted that the most effective therapeutic approach was reached in the group treated with a combination of levodopa and carbidopa, together with a higher dose of CQ10.

*M. chamomilla* L. is a commonly used plant with significant antioxidant activity because it includes flavonoids (quercetin, apigenin, luteolin, and chrysin), essential and volatile oils (alphabisabolol), bitter glucosides, and coumarin [99]. Clinical studies have confirmed that flavonoid compounds may reduce oxidative damage and neuroinflammation [100], owing to their inhibitory effects on macrophage prostaglandin E2 levels and the activity of a selective COX-2 inhibitor [101]. As oxidative damage has been linked to the pathogenesis of PD [102], Khan and colleagues investigated the potential utility of *M. chamomilla* L. tea based on chlorpromazine-induced dyskinesia in male *Wistar* rats [59]. The chlorpromazine treatment (3 mg/kg/day i.p., for 21 days) raised the cataleptic score, which was reversed by the levodopa/carbidopa combination (30 mg/kg/day i.p., for 21 days), as well as *M. chamomilla* L. tea (2.14 mL/kg/day orally, for 21 days). At the same time, the investigation of midbrain samples revealed chlorpromazine-induced gliosis, damaged, fragmented, and necrotic neurons, as well as edema, infiltration, and proliferative vessels (Table 3). All histological evidence of chlorpromazine neurotoxicity was successfully reduced by both the levodopa/carbidopa combination and *M. chamomilla* L. tea consumption.

Although initially considered safer due to extrapyramidal side effects such as Parkinsonism, second-generation antipsychotics, including risperidone, have been linked to a variety of motor impairment features in clinical trials, particularly when administered at high dosages [103]. These deleterious consequences are linked to oxidative damage in certain brain areas involved in motor control. As a result, it was unsurprising that significant effort was made to examine the possible function of antioxidant supplementation in the treatment/prevention of atypical antipsychotic-induced motor impairments. Kajero and colleagues conducted an intriguing investigation to examine the effects of the preliminary, follow-up, and simultaneous administration of cannabidiol in a risperidone model of motor disbalance in male Wistar adult rats [60]. Cannabidiol, a non-psychoactive and non-reinforcing cannabis sativa component with antioxidant, anti-inflammatory, and neuroprotective activities [104], has been shown in preclinical and clinical trials [105,106] to prevent and treat a variety of motor dysfunctions. This intriguing experimental design was carried out in such a way that the neuroprotective agent, cannabidiol (5 mg/kg/day orally), was administered prior to the risperidone (10 mg/kg/day orally, for 28 days) protocol, as well as during and after risperidone treatment, to investigate the relationship between prevention and cure. Except for the risperidone-induced increase in involuntary chewing movements (VCM test) and the decrease in extended motor coordination (rotarod test), no significant changes in motor performance were seen after the protocols were implemented. Both observed effects were alleviated by cannabidiol treatment (Table 3). More specifically, cannabidiol pretreatment improved orofacial dysfunction, whereas simultaneous administration improved endurance performance. The biochemical analysis of brain tissue samples after the antipsychotic protocol demonstrated no alterations in lipid peroxidation (MDA) or radical scavenging activities (DPPH). Interestingly, the quantification of antioxidant systems revealed a high diversity, most likely because risperidone has antioxidant properties [107].

## 5. Conclusions

Rodent experimental models may be regarded as useful for exploring new approaches for dealing with antipsychotic-induced motor dysfunction. Antioxidant supplementation demonstrated significant neuroprotective effects in the therapy of this iatrogenic dyskinesia. Considering their widespread accessibility and safety, the administration of antioxidant-rich compounds in various forms as an adjuvant therapy in long-term protocols, such as in antipsychotics in clinical practice, may benefit patients by lowering the risk of secondary Parkinsonism, attenuating it, and extending therapy. Nonetheless, despite the lower frequency of motor characteristics among adverse effects, it is clear that there are relatively few studies examining antioxidant treatments with second-generation antipsychotics. Given that their administration is also associated with various adverse effects, it seems reasonable to focus future research on that subject.

## Figures and Tables

**Table 2:** The classification of antipsychotics according to the risk of motor dysfunctions.

Risk of Motor Deficit		
High	Medium	Low
fluphenazine	chlorpromazine	All second-generation antipsychotics
trifluoperazine	thioridazine	
haloperidol		
loxapine		
pimozide		
perphenazine		
thiothixene		

**Table 3:** Antioxidant-based strategies targeting antipsychotic-induced dyskinesia in rodents.

Drug	Species and Protocol	Dyskinesia Symptoms	Pathophysiology Mechanism	Tested Substance	Antioxidant Protocol	Effect
The first-generation antipsychotics						
Haloperidol [38]	Male Wistar rats (1 mg/kg/day i.m. for 28 days)	↓ locomotor activity ↑ VCM	Neuroinflammation in striatal samples	Isoflavones: Glicitin, Genistin, Genistein, Daidzein, Daidzin, Glycitein	80 mg/kg per os for 28 days	↓ VCM ↓ IL-1β, TNF-α
Haloperidol [39]	Male Wistar rats (1 mg/kg/i.p. for 21 days)	↑ VCM ↑ tongue protrusion	Oxidative stress; neuroinflammation; apoptosis; neurotransmitters disbalance in striatal samples	Naringin (C_27_H_32_O_14_)	300 mg/kg/per os for 21 days	↓ VCM ↓ tongue protrusion ↓ oxidative stress ↓ neuroinflammation ↓ apoptosis ↑ neurotransmitters
Haloperidol [42]	Wistar rats (1 mg/kg/i.p. for 21 days)	↑ VCM ↑ tongue protrusion	Nitrosative and oxidative stress; mitochondrial dysfunction; neuroinflammation; apoptosis in striatal samples	Vitexin (C_21_H_20_O_10_)	30 mg/kg/i.p. for 21 days	↓ VCM ↓ tongue protrusion ↓ nitrosative and oxidative stress ↓ neuroinflammation ↓ apoptosis ↓ Nrf2-mediated pathway
Haloperidol [43]	Male Wistar rats (5 mg/kg/day i.p. for 28 days)	↓ locomotor activity	Oxidative stress	Quercetin (C_15_H_10_O_7_)	100 mg/kg per os for 21 days	↑ locomotion ↓ oxidative stress
Haloperidol [44]	Mice (10 mg/kg/day i.p. for 21 days)	↓ locomotor asymmetry ↓ muscle stregth	Oxidative stress; neurodegeneration	Vitamin D (C_27_H_44_O)	800 IU per os for 21 days	↑ motor performance ↓ oxidative stress ↓ neurodegeneration
				Vitamin A (C_20_H_30_O)	1000 IU per os for 21 days	
Haloperidol [45]	Male Wistar rats (0.23 mg/kg/i.p. for 14 days)	↓ locomotor activity	Oxidative stress; neuroinflammation; neurotransmitters disbalance; neurodegeneration in hippocampal samples	Cerium oxide (Ce_2_O_3_)	Nanoparticles, intranasal, 6 mg/kg	↑ locomotion ↓ oxidative stress ↓ neuroinflammation ↓ neurotransmitters disbalance ↓ neurodegeneration
Haloperidol [46]	Male Wistar rats (1.5 mg/kg/i.p. for 14 days)	↓ locomotor activity ↓ D2 receptors expression	Neurotransmitters receptors disbalance in medial brain regions	ω-3 PUFA enriched algal oil	300 mg/kg/day for 6 + 2 weeks	↑ locomotion ↑ D2 receptors expression
Haloperidol [47]	Albino mice (1 mg/kg/day i.p. for 25 days)	↓ various locomotor indicators	Oxidative stress; neurotransmitters disbalance; neurodegeneration	Rhinacanthin-C (C_25_H_30_O_5_)	5, 10, and 20 mg/kg for 25 days	↑ locomotion ↓ oxidative stress ↓ neurotransmitters disbalance ↓ neurodegeneration
Haloperidol [48]	Male albino Wistar rats (1 mg/kg/day i.p. for 14 days)	↓ locomotor activity ↓ motor coordination	Oxidative stress; neurotransmitters disbalance; neurodegeneration	Icariin (C_33_H_40_O_15_)	100 mg/kg, orally, for 14 days	↑ locomotion ↓ oxidative stress ↓ neurotransmitters disbalance ↓ neurodegeneration
Haloperidol [49]	Wistar rats (1 mg/kg/i.p. for 21 days)	↓ various locomotor indicators	Oxidative stress; neurotransmitters disbalance; neurodegeneration	Curcuminoids extract (binary and ternary inclusion complexes formulations)	15–30 mg/kg/day, orally for 21 days)	↑ locomotion ↓ oxidative stress ↓ neurotransmitters disbalance ↓ neurodegeneration
Haloperidol [50]	Wistar albino mice (5 mg/kg, infusion, for 1–7 days)	↓ locomotor activity ↓ motor coordination	α-synuclein-inclined fibrillogenesis; disturbance of neurotransmitters biosynthesis	*Datura metel* extract	50, 100, 200 mg/kg/day, per os, for 1–7 days	↑ locomotor activity ↑ motor coordination
Haloperidol [51]	Albino rats (1 mg/kg/day, i.p. for 21 days)	↓ various locomotor indicators	Oxidative stress; neurotransmitters disbalance; neurodegeneration	*Cucurbita pepo* extract	200, 400, and 600 mg/kg/day, per os for 21 days	↑ locomotion ↓ oxidative stress ↓ neurotransmitters disbalance ↓ neurodegeneration
Haloperidol [52]	Rats (1 mg/kg/day, i.p. for 21 days)	↓ various locomotor indicators	Oxidative stress; neurotransmitters disbalance; neurodegeneration	*Brassica juncea* extract	200, 400, and 600 mg/kg/day, per os for 21 days	↑ locomotion ↓ oxidative stress ↓ neurotransmitters disbalance ↓ neurodegeneration
Haloperidol [53]	Adult *Wistar* rats (0.23 mg/kg/day, i.p., for 14 days)	↓ various locomotor indicators	Not analyzed.	Bromocriptine mesylate and Glutathione	0.21 and 61.67 mg/kg/day intranasally, for 14 days, solution or nanoemulsion	↑ locomotion
Haloperidol [54]	*Wistar* rats, (1 mg/kg/day i.p., for 14 days)	↓ various behavioral indicators	Oxidative stress; neuroinflammation	Lauric acid (C_12_H_24_O_2_)	0.66 and 1.32 mg/kg, orally, for 35 days	↑ locomotion ↓ oxidative stress ↓ neuroinflammation
Haloperidol [55]	Male *Wistar* rats (1 mg/kg/day i.p., for 21 days)	↑ VCM ↑ tongue protrusion	Nitrosative and oxidative stress; mitochondrial dysfunction; neuroinflammation; apoptosis in striatal samples	Betaine (C_5_H_11_NO_2_)	30 and 100 mg/kg/day i.p., for 21 days	↓ VCM ↓ tongue protrusion ↓ nitrosative and oxidative stress ↓ mitochondrial dysfunction ↓ neuroinflammation
Haloperidol [56]	Male *Swiss* albino mice (1 mg/kg/day i.p., for 21 days)	↓ various locomotor indicators	Oxidative stress; neurotransmitter disbalance	p-coumaric acid (C_9_H_8_O_3_)	50, 75, and 100 mg/kg, orally, for 21 days	↑ locomotion ↓ oxidative stress ↓ neurotransmitter disbalance
Haloperidol [57]	*Albino Wistar* rats (0.225 mg/kg/day, i.p., for 15 days)	↓ locomotion ↑ oxidative stress	Oxidative stress	Selegiline (C_13_H_17_N) and Quercetin (C_15_H_10_O_7_)	nanoformulation (0.16 mg/kg/day, for 15 days)	↑ locomotion ↓ oxidative stress
Chlorpromazine [58]	Adult male mice (5 mg/kg/day i.p., for 21 days),	↓ various locomotor indicators	Nitrosative and oxidative stress; neurotransmitter disbalance in the brain homogenate	Co-enzyme Q10 (C_59_H_90_O_4_)	60 and 120 mg/kg, orally, for 21 days	↑ locomotion ↓ nitrosative and oxidative stress ↓ neurotransmitters disbalance
Chlorpromazine [59]	Male *Wistar* rats (3 mg/kg/day i.p., for 21 days)	↓ locomotor activity	Neurodegeneration in midbrain samples	*M. chamomilla* L. tea	2.14 mL/kg/day orally, for 21 days	↑ locomotor activity ↓ neurodegeneration
The second-generation antipsychotics						
Risperidone [60]	Male *Wistar* adult rats (10 mg/kg/day orally, for 28 days)	↑ VCM ↓ endurance performance	Alterations in antioxidant capacity	Cannabidiol (C_21_H_30_O_2_)	5 mg/kg/day orally	↓ VCM ↑ endurance performance complex response on oxidative equilibrium
