# Correction: Velickovic et al. The Advances in Antipsychotics-Induced Dyskinesia Rodent Models: Benefits of Antioxidant Supplementation. *Biomedicines* 2025, *13*, 512

**DOI:** 10.3390/biomedicines13092216

**Published:** 2025-09-10

**Authors:** Uros Velickovic, Dragica Selakovic, Nemanja Jovicic, Marina Mitrovic, Vladimir Janjic, Sara Rosic, Suzana Randjelovic, Dragan Milovanovic, Gvozden Rosic

**Affiliations:** 1 Department of Physiology, Faculty of Medical Sciences, University of Kragujevac, 34000 Kragujevac, Serbia; uvelickovic26@gmail.com (U.V.);; 2 Department of Histology and Embryology, Faculty of Medical Sciences, University of Kragujevac, 34000 Kragujevac, Serbia; 3 Department of Medical Biochemistry, Faculty of Medical Sciences, University of Kragujevac, 34000 Kragujevac, Serbia; 4 Department of Psychiatry, Faculty of Medical Sciences, University of Kragujevac, 34000 Kragujevac, Serbia; 5 Psychiatry Clinic, University Clinical Center Kragujevac, 34000 Kragujevac, Serbia; 6 Department of Emergency Medicine, University Clinical Center Kragujevac, 34000 Kragujevac, Serbia; 7 Department of Pharmacology and Toxicology, Faculty of Medical Sciences, University of Kragujevac, 34000 Kragujevac, Serbia

The authors would like to make the following corrections to this published paper [1]:

Instead of the following retracted Ref. [24] in the paper:

Taylor, D.M.; Velaga, S.; Werneke, U. Reducing the stigma of long acting injectable antipsychotics—Current concepts and future developments. *Nord. J. Psychiatry* **2018**, *72* (Suppl. S1), S36–S39.

We have now included the following [2]:

Nadesalingam, N.; Chapellier, V.; Lefebvre, S.; Pavlidou, A.; Stegmayer, K.; Alexaki, D.; Gama, D.B.; Maderthaner, L.; von Känel, S.; Wüthrich, F.; et al. Motor abnormalities are associated with poor social and functional outcomes in schizophrenia. *Comp. Psychiatry* **2022**, *115*, 152307.

There are no changes to the order of references. The authors state that the scientific conclusions are unaffected. This correction was approved by the Academic Editor. The original publication has also been updated.
